# Significance of the ‘line sign’ in the diagnosis of congenital imperforate anus on prenatal ultrasound

**DOI:** 10.1186/s12887-021-03084-2

**Published:** 2022-01-03

**Authors:** Chan Yin, Lili Tong, Dan Nie, Zhihui Fei, Xiaoqun Tan, Mingxiang Ma

**Affiliations:** The Maternal and Child Health Hospital of Changde city, Changde, 415000 Hunan China

**Keywords:** Prenatal ultrasound diagnosis, Congenital imperforate anus, The ‘target sign’, The ‘line sign’

## Abstract

**Background:**

The prenatal diagnosis of foetal imperforate anus is difficult. Most previous studies have been case reports. To provide useful information for diagnosing foetal imperforate anus, a retrospective review of diagnostic approaches was conducted. Ultrasonography was performed in 19 cases of foetal imperforate anus from 2016 to 2019 at our prenatal diagnostic centre. The prenatal sonographic features and outcomes of each case were collected and evaluated.

**Result:**

The anal sphincter of a normal foetus shows the ‘target sign’ on cross-sectional observation. Of the 19 cases of imperforate anus, 16 cases were diagnosed by the ultrasound image feature called the ‘line sign’. 1 case with tail degeneration was low type imperforate anus with the irregular ‘target sign’ not a real ‘target sign’. There was two false-negative case, in which the ‘target sign’ was found, but irregular.

**Conclusion:**

In this study, we find that the anus of a foetus with imperforate anus presents a ‘line sign’ on sonographic observation. The absence of the ‘target sign’ and then the presence of the ‘line sign’ can assist in the diagnosis of imperforate anus. The ‘line sign’ can be used as a secondary assessment to determine the type of the malformation following non visualization of the ‘target sign’. The higher the position of the imperforate anus is, the more obvious the ‘line sign’. It is worth noting that the finding of the short ‘line sign’ and irregularr ‘target sign’ can not ignore the low type imperforate anus.

## Introduction

Imperforate anus is a common congenital malformation that is caused by hindgut development defects or retardation. Imperforate anus occurs in approximately 1 per 5000 live births [1]. The deformity can occur alone or with other congenital malformations or chromosomal abnormalities. The prenatal diagnosis of imperforate anus is difficult, as there are no direct ultrasound signs. The lack of the ‘target sign’ is often used for predictive diagnosis. In previous studies, the diagnosis of imperforate anus by prenatal ultrasound was based on indirect sonographic features, such as dilatation of the proximal bowel with atresia and the enterolith sign [1–3]. However the prenatal detection rate of anal atresia is yet low (8.2%) [4].

Prenatal diagnosis of imperforate anus is not always possible. Nevertheless, consciousness of the condition and the ability to recognize the most typical ultrasound findings in imperforate anus may improve the detection rate. This study compared sonographic features of the anus between normal foetuses and foetuses with imperforate anus. The aim of this study was to describe the imaging features of imperforate anus by prenatal ultrasound. The secondary aim was to explore whether the ‘line sign’ can be used as a secondary assessment to determine the type of the malformation following non visualization of the ‘target sign’.

## Materials and methods

### Patients

In this study, among 16,475 pregnant women who underwent prenatal ultrasound examination at the Maternal and Child Health Hospital of Changde city from January 2016 to February 2019, there were 19 foetuses with imperforate anus. The Maternal and Child Health Hospital of Changde city is the Prenatal Diagnosis Center in the area. Some fetuses highly suspected of imperforate anus were referred to our center for diagnosis from other hospitals. All cases of imperforate anus were confirmed by postnatal examination or autopsy. The study was approved by the ethics committee of the hospital. Medical abortion was the methodology for legal termination of pregnancy. It was accomplished following hospital admission and signed informed consent.

### Equipment and methods

The equipment used for examinations in the study were a GE Voluson E8 system and a Philips A70 colour Doppler ultrasound diagnostic instrument. The specific sonographic marker for imperforate anus was assessed using conventional two-dimensional (2D) real-time ultrasound. And then we used three-dimensional (3D) to visualize the fetal anal canal. Two doctors (Lili Tong and Zhihui Fei) with more than 10 years of experience performed the examinations, analysed the cases independently and provided a diagnosis. In the case of conflicting results, a superior doctor (Chan Yin) was consulted to come to a unified conclusion.

All data processing was performed using the statistical software package SPSS 23.0.

### Inspection standards

Foetuses at 21–24 weeks and 30–34 weeks were examined for anal deformity. We needed to obtain a cross-sectional image of the anus by ultrasound examination. After the foetal bladder cross-section appeared, the ultrasound beam was gradually moved parallel to the foetal caudal direction. Before the disappearance of the skin of the buttocks, a cross-sectional image of the anus between the buttocks was displayed. At this time, we studied the characteristics of the image. It should be noted that the gluteal sulcus of the foetus should be perpendicular to the direction of the acoustic beam when the images are captured.

**Fig. 1:**
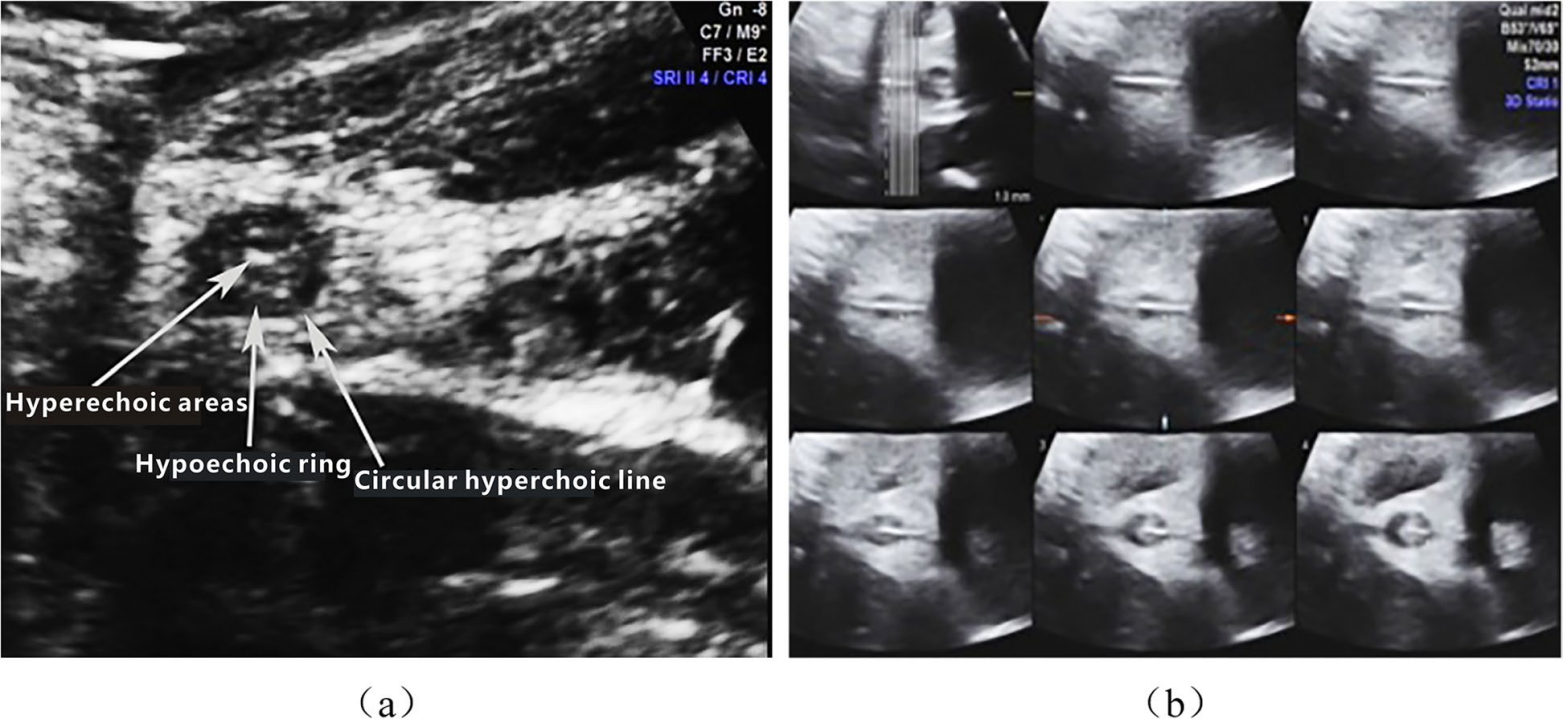
**a** The anal sphincter of normal foetuses show the ‘target sign’ on cross section, ‘high-low-high’ concentric circle echo. **b** The ‘target sign’ with TUI mode

The anal sphincter of a normal foetus showed the ‘target sign’ on cross-sectional sonographic observation. The ‘target sign’ showed three structural layers, resulting in a ‘high-low-high’ concentric circle echo. The hyperechoic areas in the centre were the mucosa of the anal canal. The thick round hypoechoic ring was the anal sphincter. The most peripheral area was a circular hyperechoic line, which was a reflection of the interface between the outer layer of the anal sphincter and the surrounding tissue [5] (Fig. 1).

## Results

During the 3-year study period, through the ultrasound examination of 16,475 pregnant women, 19 fetuses presenting with imperforate anus were prospectively evaluated for the absence of the ‘target sign’ and the presence of the ‘line sign’. Table 1 shows the relevant sonographic features in these cases. There were 13 (68.4%) males and 6 (31.6%) females among the 19 cases of imperforate anus. There were 18 (94.7%) cases of non-dilatation and 1 (5.3%) case of dilatation. There were 3 (15.8%) cases of polyhydramnios, 1 (5.3%) cases of oligohydramnios and 15 (78.9%) cases of a normal amniotic fluid index.

**Table 1:** The relevant sonographic features in 19 cases

Characteristics	Number of fetuses (%)
Sex	
Male	13 (68.4)
Female	6 (31.6)
Amniotic fluid index	
Polyhydramnios	3 (15.8)
Oligohydramnios	1 (5.3)
Normal amniotic fluid index	15 (78.9)
Bowel dilatation	
Non-dilatation	18 (94.7)
Dilatation	1 (5.3)
Isolated imperforate anus	3 (15.8)
Associated perineal / genitalia malformations	13 (68.4)
VACTERL syndrome	5
OEIS complex	3
Urorectal septal sequence syndrome	2
Caudal regression syndrome	3
Other malformations	3 (15.8)
Right auricle malformations	1
Gallbladder agenesis	1
Horseshoe kidney with a single umbilical artery	1

There were 3 (15.8%) cases of simple imperforate anus and 16 (84.2%) cases of imperforate anus with other malformations. The other malformations included 5 cases of VACTERL syndrome, 3 cases of OEIS complex, 2 cases of urorectal septal sequence syndrome, 3 cases of caudal regression syndrome, 1 case of malformation of the right auricle, 1 case of gallbladder agenesis and 1 case of horseshoe kidney with a single umbilical artery. Sonographic findings were compared with clinical results, as shown in Table 2.

**Table 2:** Sonographic findings and clinical results in imperforate anus

Case	Age	Gestational age at diagnosis (weeks)	Sex	Sonographic Finding				Pathologic and Surgical Findings		
				Amniotic fluid index (cm)	Intestinal dilatation	Diagnosis	Other Anomalies	Fetal Outcome	diagnosis	Other Anomalies
1	32	26	M	25.2	No	Imperforate anus	VACTERL syndrome	LTOP	Imperforate anus	VACTERL syndrome
2	39	26	F	12.9	No	Imperforate anus	Caudal regression syndrome	LTOP	Imperforate anus	Caudal regression syndrome
3	31	24	F	15.9	No	Imperforate anus	OEIS complex	LTOP	Imperforate anus	OEIS complex
4	30	33	M	15.0	No	Imperforate anus	Horseshoe kidney, single umbilical artery	LTOP	Imperforate anus	Horseshoe kidney, single umbilical artery
5	40	33	F	1.2	No	Imperforate anus	Caudal regression syndrome	LTOP	Imperforate anus	Caudal regression syndrome
6	26	26	M	18.9	No	Imperforate anus	VACTERL syndrome	LTOP	Imperforate anus	VACTERL syndrome
7	35	32	M	11.4	No	Imperforate anus	...	LTOP	Imperforate anus	...
8	27	24	M	11.1	No	Imperforate anus	OEIS complex	LTOP	Imperforate anus	OEIS complex
9	36	26	M	17.7	No	normal	...	Alive, surgical treatment	Imperforate anus	...
10	25	27	M	5.6	Yes	Imperforate anus	Urinary rectal septum	LTOP	Imperforate anus	urorectal septal sequence syndrome
11	33	23	F	12.8	No	Imperforate anus	Urinary rectal septum	LTOP	Imperforate anus	urorectal septal sequence syndrome
12	26	25	F	8.8	No	Imperforate anus	VACTERL syndrome	LTOP	Imperforate anus	VACTERL syndrome
13	26	26	M	9.2	No	Imperforate anus	OEIS complex	LTOP	Imperforate anus	OEIS complex
14	36	27	M	25.8	No	Imperforate anus	VACTERL syndrome	LTOP	Imperforate anus	VACTERL syndrome
15	23	25	M	15.9	No	Imperforate anus	VACTERL syndrome	LTOP	Imperforate anus	VACTERL syndrome
16	28	30	M	10.1	No	Imperforate anus	Gallbladder agenesis	Alive, surgical treatment	Imperforate anus	Gallbladder agenesis
17	29	23	M	13.7	No	Imperforate anus	Malformation of right auricle	LTOP	Imperforate anus	Malformation of right auricle
18	22	22	M	23.2	No	normal	...	Alive, surgical treatment	Imperforate anus	...
19	36	26	M	15.3	No	Imperforate anus	Caudal regression syndrome	LTOP	Imperforate anus	Caudal regression syndrome

Among the 16,475 fetuses in which the regular the ‘line sign’ was identified prenatally, no cases of imperforate anus were reported postnatally or at the time of postmortem examination. Of the 19 cases of imperforate anus, 16 cases were diagnosed by the ultrasound image feature called the ‘line sign’. 1 case with tail degeneration was low type imperforate anus with the irregular ‘target sign’ not a real ‘target sign’. There was two false-negative case, in which the ‘target sign’ was found, but irregular. Both cases were found to be isolated low imperforate anus after delivery. Overall, in our study, absent the ‘target sign’ and present of the ‘line sign’ on prenatal sonography had false negative rate of 10.5% and no false positive cases for the diagnosis of imperforate anus.

In 16 cases, we were able to confidently demonstrate the imperforate anus by identifying the absence of the ‘target sign’ and the presence of the ‘line sign’. There was one case of low type imperforate anus with caudal regression syndrome. Postmortem examination of the fetus showed no anal canal. The distance between the blind end of rectum and anal skin was 0.5 cm. Pathology showed the existence of anal column, anal mucosa, irregular internal sphincter and external sphincter. The development of anal canal in low type imperforate anus was closer to normal.

The results of serological screening were normal in 19 cases. Non-invasive prenatal testing (NIPT) was performed in 6 cases. The result was normal in 5 cases and abnormal in 1 case. In the abnormal case, 18q22.1 - q23.79 m deletion and 11.79 mb microduplication were detected.

## Discussions

The prenatal diagnosis of imperforate anus is difficult. However, the diagnosis may be suspected by the sonographic observation of colon dilatation in or beyond the second trimester or by the absence of a typical anal sonographic appearance in the third trimester. Often, the diagnosis is only made after birth [6, 7]. Most previous studies have been case reports [8].

Lianli et al. reported that imperforate anus can be diagnosed according to the area of the anal sphincter, the distance between the anal sphincter and ischia, absence of the anal canal and the anterior and posterior size of the rectum [9]. Brantberg et al. reported the prenatal sonographic diagnosis of imperforate anus relied on indirect findings, such as abnormally dilated distal bowel segments in early second trimester or the presence of calcified intraluminal meconiumor enterolithiasis in the second and third trimesters [1]. However, our study found that bowel dilatation is not present in most cases of imperforate anus, and there are no signs of enterolith or hydramnios. Therefore, it is difficult to diagnose imperforate anus with the above methods.

Ying et al. studied the cross-sectional features of the anal sphincter in normal foetuses on ultrasound examination (‘target sign’) [5]. Few studies have summarized the sonographic features of the anal sphincter in foetuses with imperforate anus. Ochoa et al. have found that in a high-risk population, the absence of perianal muscular complex (PAMC) seems to be a highly sensitive and specific sonographic marker for anorectal atresia. But the role of routine sonographic identification of the PAMC for cases of isolated imperforate anus remains to be determined [10]. In this study, by analysing a large number of ultrasound image features of the foetal anus, we found a typical ultrasound image feature of imperforate anus (‘line sign’).

**Fig. 2:**
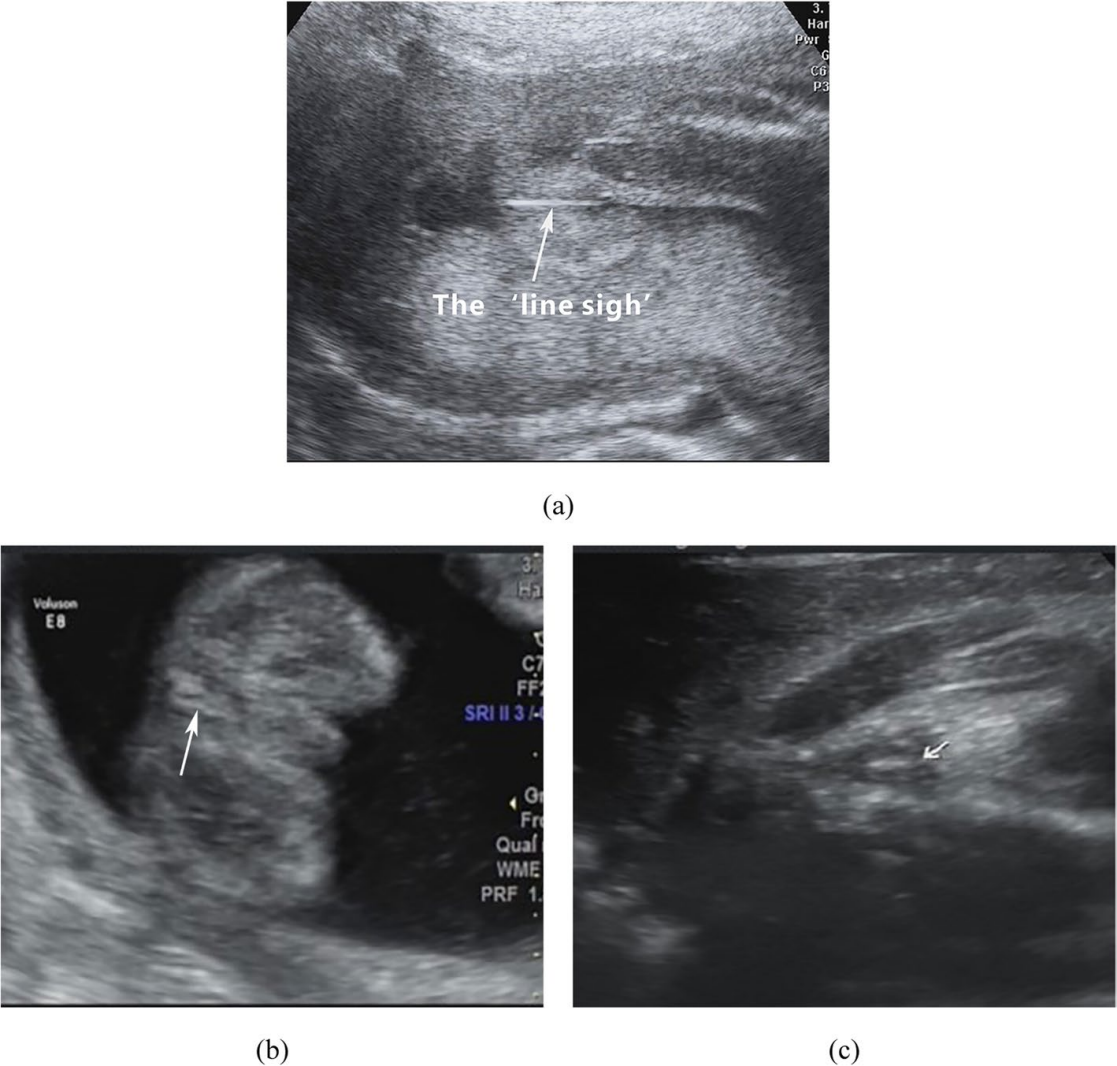
The sonographic demonstration of the anus of the fetus with imperforate anus showed ‘line sign’. The higher the position of the imperforate anus was, the more obvious the ‘line sign’. **a** High type imperforate anus. **b** Imperforate anus between high and low position. **c** Low type imperforate anus

In this study, we found that sonographic observation of the imperforate anus revealed the ‘line sign’ (Fig. 2a), with no hyperechoic area or round hypoechoic ring. The reflection of the interface between the outer layer of the anal sphincter and surrounding tissues was absent, and the area of the anus was completely covered with skin. When the direction of the ultrasound beam was perpendicular to the gluteal sulcus, a hyperechoic line formed in the area of the anus. We denoted this sonographic feature as the ‘line sign’. Therefore, we regarded the ‘line sign’ formed when the direction of the ultrasound beam was perpendicular to the gluteal sulcus as the diagnostic standard for imperforate anus. After autopsy, we also found that the higher the position of the imperforate anus was, the more obvious the ‘line sign’ (Fig. 2). There was no anal canal. The anal sphincter was absent or maldeveloped.

**Fig. 3:**
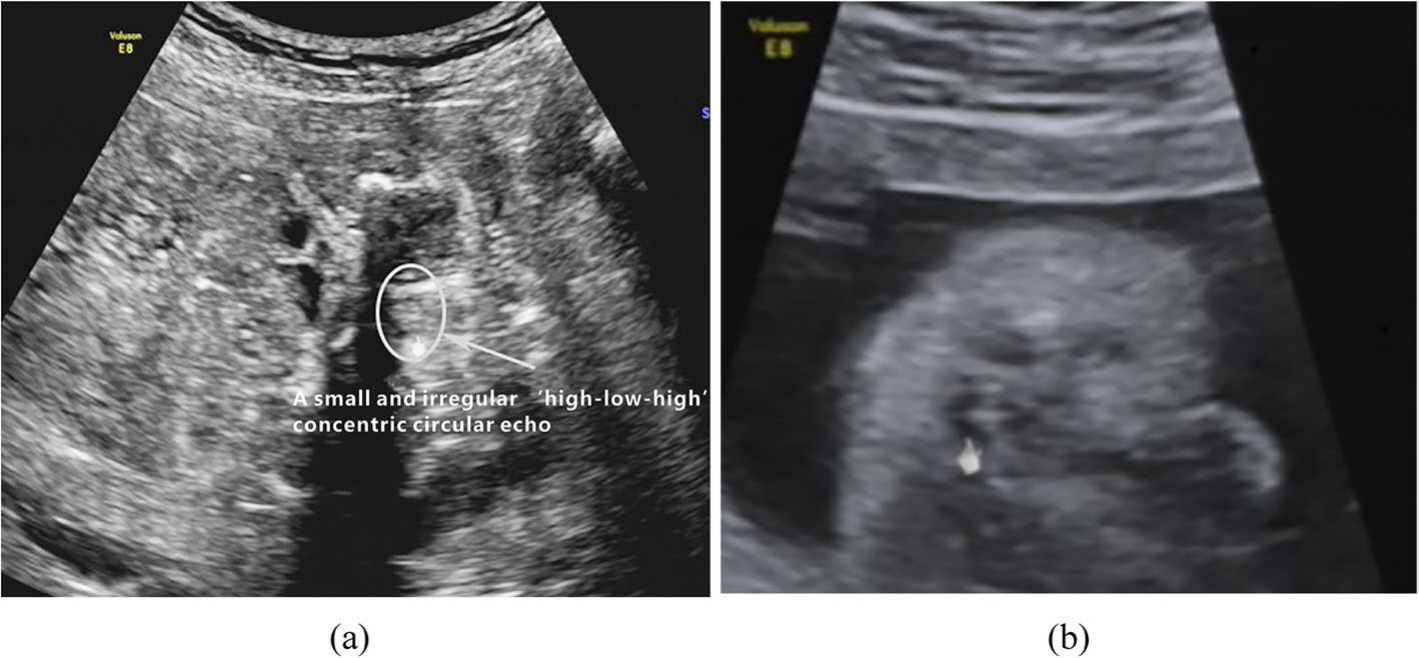
Low type imperforate anus, a small and irregular ‘high-low-high’ concentric circular echo similar to ‘target sign’

**Fig. 4:**
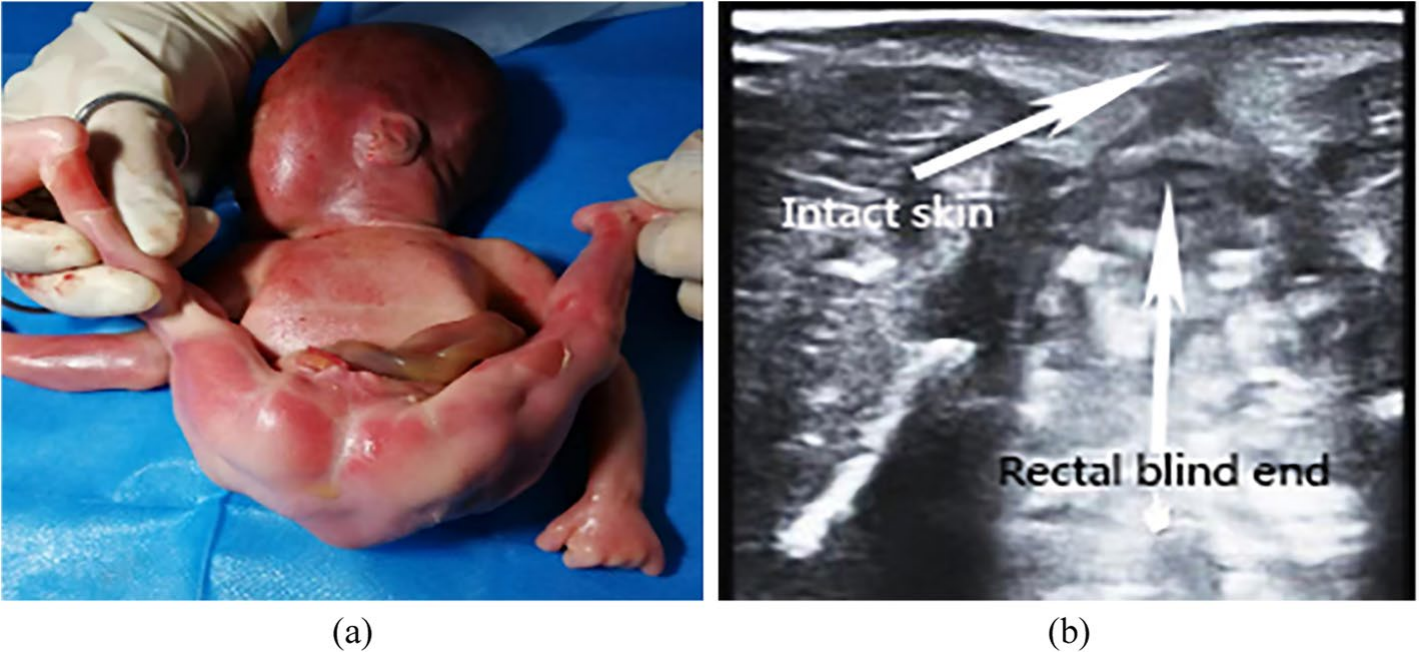
**a** Imperforate anus. **b** Low type imperforate anus, the structure of intestinal wall in the blind end of rectum

Rohrer et al. pointed out that the absence of the ‘target sign’ is the foremost direct imaging sign suggesting anorectal malformation [11]. However, we found that there was a irregular ‘target sign’ in low imperforate anus. Imperforate anus can be divided into high type and low type depending on the relation-ship between the distal rectal pouch and the puborectalis muscle [12]. Haber et al. have shown that the prenatal diagnosis of low type imperforate anus on ultrasound is very difficult [13, 14]. There were 3 cases of low type imperforate anus in our study. Prenatal examination showed no bowel dilatation. There were a short ‘line sign’ easily overlooked and a irregular ‘high-low-high’ concentric circular echo, which looked like the ‘target sign’ (Fig. 3). There were two layers of smooth muscle in the muscular layer of the rectal wall, one for the inner ring and one for the outer longitudinal layer. This was consistent with the arrangement of the sphincter of the anal canal. Therefore, there was also a hypoechoic ring on sonographic observation. The rectal wall was thinner than the sphincter of the anal canal. Therefore, the hypoechoic ring formed by the rectal wall was thinner than that of the ‘target sign’. After the induction of labour, a sagittal scan with a high-frequency probe clearly showed the intestinal wall structure of the blind end of the rectum (Fig. 4). In general, the ‘high-low-high’ concentric circle echo formed by low type imperforate anus was different from that formed in normal foetuses (‘target sign’).

**Fig. 5:**
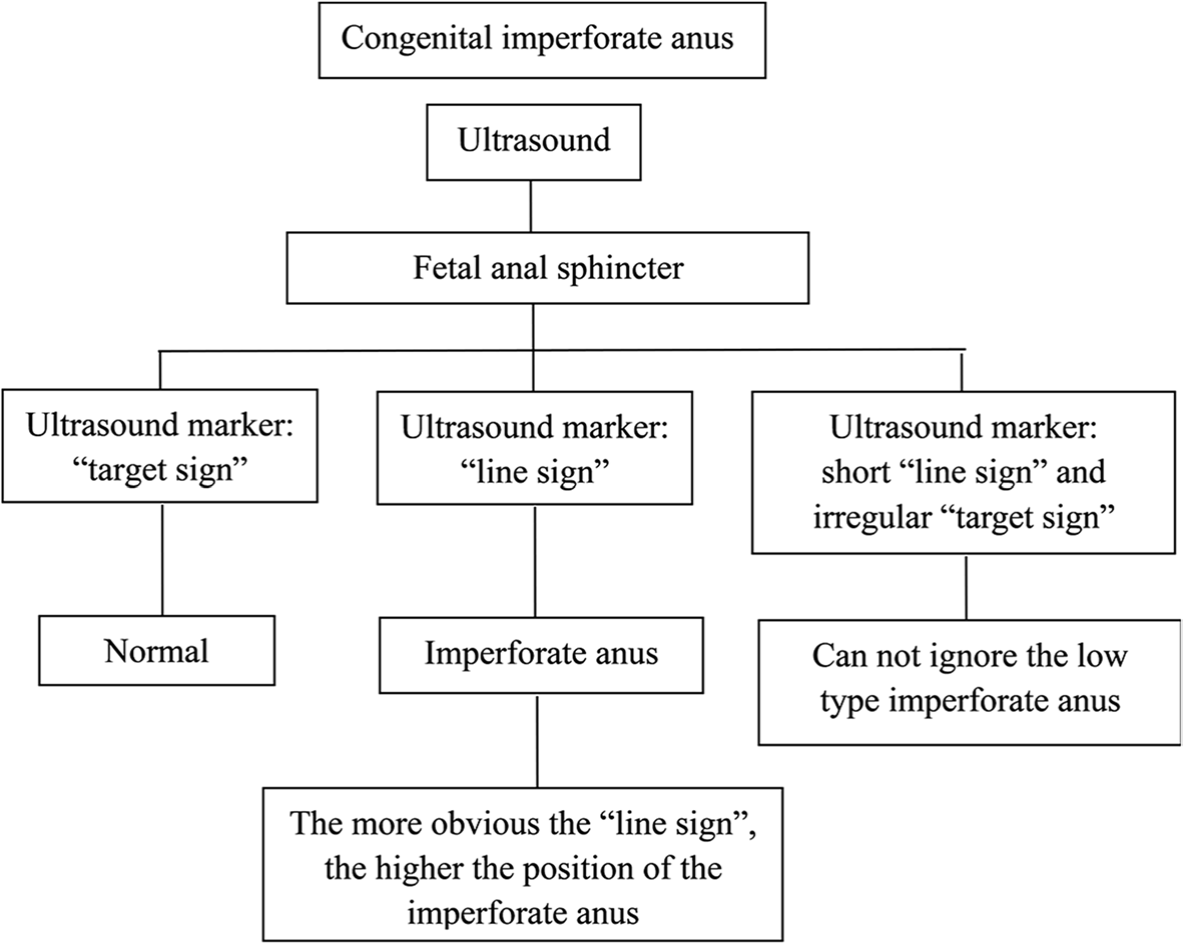
Clinical-diagnostic flowchart to be used in the prenatal management of congenital imperforate anus

Following the analysis of the medical records and results from our series, we developed a clinical-diagnostic flowchart to be used in cases of prenatally detected congenital imperforate anus (Fig. 5) .

## Conclusions

The ability to recognize the most typical ultrasound findings in imperforate anus can improve the detection rate. The absence of the ‘target sign’ and then the presence of the ‘line sign’ can assist in the diagnosis of imperforate anus. The ‘line sign’ can be used as a secondary assessment to determine the type of the malformation following non visualization of the ‘target sign’.

The higher the position of the imperforate anus is, the more obvious the ‘line sign’. It is worth noting that the finding of the short ‘line sign’ and irregular ‘target sign’ can not ignore the low type imperforate anus. Several additional limitations apply to our study. These include that the small sample size of low type imperforate anus, and we didn’t explore the role of 2D vs. 3D ultrasound with regard to the prenatal diagnosis of the imperforate anus.

## Data Availability

The data analysed during this study are included in the tables in this published article. The datasets used during the current study are available from the corresponding author on reasonable request.

## Abbreviations

TUI: Tomography ultrasonography imaging
LTOP: Legal termination of pregnancy
M: Male
F: Female
PAMC: Perianal muscular complex
